# A Comparison of the Shear Bond Strength between a Luting Composite Resin and Both Machinable and Printable Ceramic–Glass Polymer Materials

**DOI:** 10.3390/ma17194697

**Published:** 2024-09-25

**Authors:** Nazli Aydin, Selin Celik Oge, Ogulcan Guney, Onur Okbaz, Yasar Sertdemir

**Affiliations:** 1 Department of Prosthodontics, Faculty of Dentistry, Cukurova University, Adana 01250, Turkey; selincelik@cu.edu.tr; 2 The Abdi Sutcu Vocational School of Health Services, Cukurova University, Adana 01790, Turkey; 3 Faculty of Dentistry, Cukurova University, Adana 01250, Turkey; ogulcanguney04@gmail.com (O.G.); onurokbaz98@gmail.com (O.O.); 4 Department of Biostatistics and Medical Informatics, Faculty of Medicine, Cukurova University, Adana 01790, Turkey; yasarser@cu.edu.tr

**Keywords:** three-dimensional printing, 3D printed permanent resin, printable permanent resin, ceramic glass polymer materials, shear bond strength, self-adhesive luting composite

## Abstract

This study aims to compare the shear bond strength (SBS) and Weibull characteristics between a luting composite resin and both printable and two different machinable ceramic–glass polymer materials. A total of 36 substrates were prepared, with 12 in each group. Printable substrates (12 mm × 12 mm × 2 mm) were printed by using permanent crown resin (3D-PR). Machinable substrates were obtained from Cerasmart 270 (CS) and Vita Enamic (VE) blocks (2 mm in thickness). The bonding surfaces of substrates were polished and airborne abraded (50 µm Al_2_O_3_). A self-adhesive luting composite resin (RelyX U200, 3M ESPE, St. Paul, MN, USA, SLC) was applied on substrates with the help of a cylindrical (Ø3 × 3 mm) mold. The SBS test was conducted using a universal test machine. The SBSs of three materials were compared using a one-way analysis of variance (ANOVA) (*α* = 0.05). The Weibull modulus was calculated for each material. The Kruskal–Wallis and chi-square tests were carried out for the failure mode analysis. There was no significant difference between the SBSs of the three materials (*p* = 0.129). The Weibull modulus was 3.76 for the 3D-PR, 4.22 for the CS, and 6.52 for the VE group. Statistical analysis showed no significant difference between the failure modes of the groups (*p* = 0.986). Mixed-failure fractures were predominantly observed in all three groups. The results show that the SBS of the SLC to printable 3D-PR is comparable to that of CS and VE material. Failure modes of printable 3D-PR show similar results with two different machinable ceramic–glass polymers.

## 1. Introduction

Chairside computer-aided design and computer-aided manufacturing (CAD/CAM) technology and CAD/CAM dental materials have been evolving and diversifying [1,2,3,4,5]. Currently, ceramics and composites are available esthetic materials in this field, and they have many advantages and disadvantages [1,3,5]. Therefore, ceramic–glass polymer materials were developed to combine the benefits of ceramics and composites, such as lower abrasive effects, durability, ease of fabrication, polishability, and intraoral reparability because of their resin content [2,6]. Polymer-infiltrated-ceramic network materials (VITA Enamic) (VE) and force-absorbing hybrid ceramics (Cerasmart 270) (CS) are commercially available machinable ceramic–glass polymer materials [5,7,8,9,10,11]. These materials are highly attractive for dental practice, as they are industrially polymerized blocks with no need for any post-milling processes such as firing or curing [5,7].

Additive manufacturing (AM) is also a part of CAD/CAM technology, along with subtractive manufacturing (SM) [12,13,14,15]. AM saves material as it only uses the amount of the definitive product and can produce more complex geometries, unlike the SM [16,17]. Furthermore, the manufacturers recommend that the built object require a post-processing step (such as cleaning with alcohol and post-polymerization) in order to stabilize the mechanical and biological properties [12,14,18]. One of the printable ceramic–glass polymer materials, VarseoSmile Crown Plus (3D-PR), has recently been developed for permanent restorations that can be printed in one session [15,19,20,21].

The strength and durability of the bond between the restoration and luting agent are one of the most crucial factors for the restoration’s success [3,8,9,18,22,23,24]. Ideally, luting composite resins are suggested for long-term successful restorations [3,4,8,25,26]. Self-adhesive luting composite resins (SLCs) have commonly been used as modern luting agents that eliminate the preprocessing of teeth tissue [4,22,26,27]. The adhesion of SLC to tooth structure has been clarified and documented in great detail in previous studies [22,26,28]. However, the factors affecting the restoration side are also important. The susceptibility of the bonding surface to physical or chemical modification is determined by the type of material and fabrication technique, such as casting, pressing, sintering, and machining [3,29].

In the literature, the mechanical and optical properties of 3D-PR have been compared with machinable ceramic–glass polymer materials, but no studies have compared the bond strengths with an SLC [15,20]. There are many laboratory studies evaluating the bond strengths of SLC to machinable ceramic–glass polymer materials [4,6,9,23,30]. The in vitro comparison of a printable ceramic–glass polymer material with machinable ones can be a useful method for pre-estimating the clinical performance of bond strength. Prior to clinical dental applications, Weibull statistics are important for evaluating the reliability of bond strength tests and understanding specimens’ structural reliability and strength properties [26,31,32,33]. Whereas limited data are available on the bond strength of 3D printed temporary resin to luting composite resins, the authors are unaware of previous research on 3D-PR [18,34].

The purpose of this study is to compare the shear bond strengths (SBSs) of an SLC to printable 3D-PR and two different machinable ceramic–glass polymers, VE and CS; and then evaluate the Weibull distribution of tested materials. The null hypothesis stated that there would be no difference in terms of the SBS of the SLC to the printable 3D-PR, and machinable VE or CS.

## 2. Materials and Methods

According to our power analysis, to detect a difference of MPa ≥ 2 with 80% power and a 0.05 significance level at a standard deviation of 2.5, a total of 36 samples, 12 per group, were calculated. Table 1 describes the information about the materials used in this study. Randomization was performed using a web-based, free tool (research randomizer Version 4.0, access date: 8 January 2021, http://www.randomizer.org) to eliminate bias across groups in specimen selection. This in vitro study required no ethical approval.

### 2.1. Preparation of Substrates

Using CAD software (free version, access date: 4 December 2021, https://www.blender.org; Blender Foundation, The Netherlands, Amsterdam), 3D-PR substrates with dimensions of 12 mm × 12 mm × 2 mm were designed and exported as a standard tessellation language (STL) file. According to the STL data, twelve 3D-PR substrates were printed by using a 3D printer (Form 3B; Formlabs, Somerville, MA, USA) (90 degrees printing orientation, LED, λ = 405 nm, the layer thickness of 50 μm). After the printing was complete, the substrates were immersed in isopropyl alcohol (IPA ≥ 99%) for 3 min in a washer (Form Wash; Formlabs, Somerville, MA, USA). Then, they were polymerized in a unit (Form Cure; Formlabs, Somerville, MA, USA) at 60 °C for 20 min. After the supports had been eliminated, the second polymerization was carried out for 20 min.

Next, 2 mm thickness machinable VE and CS substrates were cut from each block using a water-cooled cutting instrument (Accutom-10; Struers, OH, USA). Subsequently, all substrates were embedded in autopolymerized acrylic resin (Imicryl SC; Imicryl Dental Materials, Konya, Turkey) with a silicone mold (3 cm × 2.5 cm × 2 cm) (Presigum; President Dental GmbH, Germany). The bonding surfaces were polished with silicon carbide abrasive papers (800- and 1200-grit) to standardize them. Then, each substrate underwent 50 µm Al_2_O_3_ airborne particle abrasion (Korox 50; Bego, Bremen, Germany) for 15 s at a pressure of 0.1 MPa at a distance of 10 mm. After ultrasonically cleaning with distilled water, they were air-dried.

### 2.2. Application of Luting Composite Resin

After machinable and printable substrates were prepared, the SLC was mixed and filled to the top of the cylindrical silicone mold (Ø3 × 3 mm) (Presigum; President Dental GmbH, Germany). The light power intensity of the light polymerization unit (Rainbow LED Curing Light, Liang Ya Dental Equipment Co., Ltd., Guangzhou, China) was measured to 950 mW/cm^2^ (Demetron LED Radiometer, SDS Kerr, Middleton, WI, USA). The top surfaces of the specimens were polymerized for 20 s. After removing the silicone mold, the specimens were polymerized on the four proximal sides and the top for 20 s each in order to polymerize them completely. Before testing for SBS, every specimen was submerged in distilled water to 37 ± 2 °C for 24 ± 2 h.

### 2.3. Shear Bond Strength (SBS) Test

**Figure 1:**
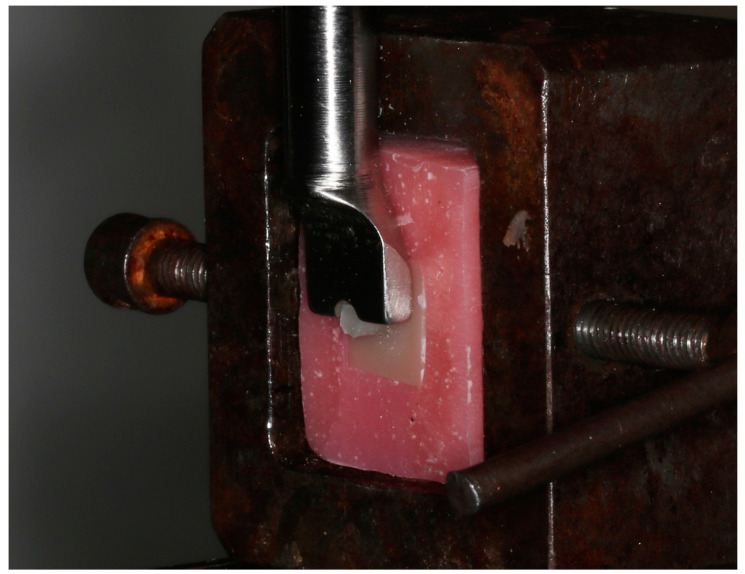
Shear bond strength test design.

A universal test machine (Testometric M500-25AT; Testometric Co., Ltd., Rochdale, UK) was used to perform the macro-SBS test at the crosshead speed of 1 mm/min until the specimens failed (Figure 1). A notch-shaped rod was used to apply the testing load. The bonding interface was parallel to the loading direction. Failure loads were recorded, and SBS values of the specimens were calculated using the formula: SBS = $\frac{L}{A}$, where *L* is the load at failure (Newton), and *A* is the specimen’s bonding area (mm^2^). The mean SBS was calculated for each study group.

### 2.4. Microscopic Characterization of Failure Modes

**Figure 2:**
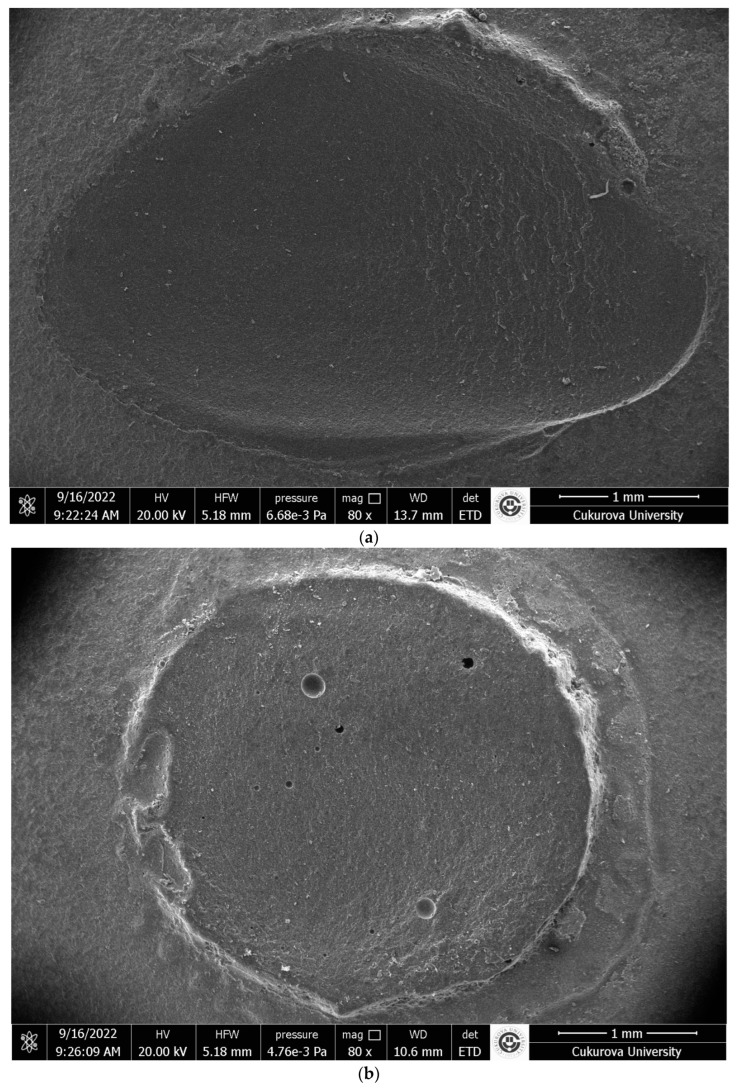

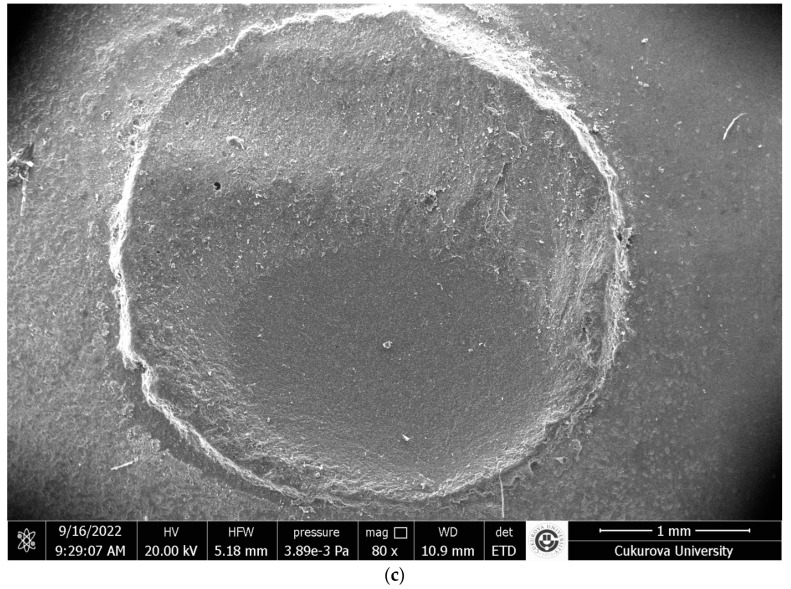
(**a**) Cohesive failure in restorative material, scanning electron microscope image (×80 magnification). (**b**) Cohesive failure in luting composite, scanning electron microscope image (×80 magnification). (**c**) Mixed failure, scanning electron microscope image (×80 magnification).

The failure modes were analyzed by using an optical microscope (×40 Zeiss Primostar; Carl Zeiss, Germany) after the SBS test was completed. Failure modes were categorized as (1) adhesive, failure at the bonding line, no remnants from SLC; (2) mixed, failure line comprises both restorative material and luting composite (partially restorative material, partially SLC visible); (3) cohesive failure in luting composite, fracture surface consists of only SLC; (4) cohesive failure in restorative material, fracture surface consists of only restorative material. The scanning electron microscope (SEM) (FEI; Quanta 650 FEG, Salem, OR, USA.) was used to acquire representative photographs of failure modes at a magnification of ×80 (Figure 2a–c).

### 2.5. Statistical Analysis

A statistical software program (IBM SPSS v20.0; IBM Corp., Armonk, NY, USA) was used for the statistical analysis of the collected data. The Levene test showed that the data were normally distributed. In addition to the descriptive analysis, a 1-way analysis of variance (ANOVA) was used for evaluating the SBS measurements (*α* = 0.05). The Kruskal–Wallis test and chi-square test were used for the failure mode analysis. In addition, a Weibull analysis was conducted and Weibull modulus’ were calculated [31].

## 3. Results

### 3.1. Shear Bond Strength (SBS) and Weibull Modulus

**Figure 3:**
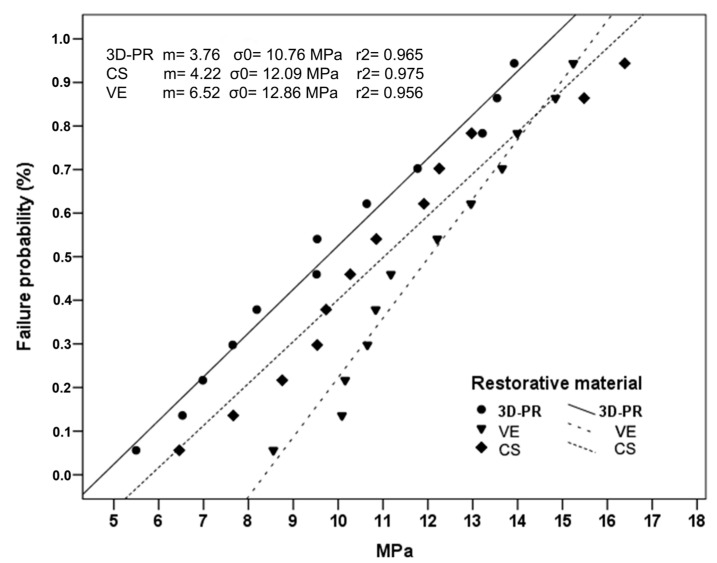
Weibull plots of tested ceramic–glass polymer materials.

Table 2 represents the mean SBSs and standard deviations for all tested groups. The highest mean SBS value (12.03 ± 2.11 MPa) was observed in the VE, and the lowest mean SBS value (9.74 ± 2.88 MPa) was observed in the 3D-PR, among the tested groups. Statistically, the variance was homogeneous among groups (*p* = 0.529). There was no significant difference among the three groups (ANOVA, *p* = 0.129). In accordance with the Weibull analysis (Figure 3), the highest Weibull modulus was observed for VE (*m* = 6.52) followed by CS (*m* = 4.22), and the data for the machinable ceramic–glass polymer materials were distributed uniformly. Although the lowest Weibull modulus was seen for the 3D-PR (*m* = 3.76), a printable ceramic–glass polymer was also observed to have a homogeneous distribution.

### 3.2. Distribution of the Failure Modes

Distribution of the failure modes was also examined, and there was no significant difference between the three groups (Table 3). Mixed-failure fractures were predominantly observed in all groups. Adhesive failure mode was not observed in any groups. When the relationship between failure modes and SBS is examined, it was seen that the mean SBS of the specimens showing a cohesive failure in luting composite was significantly lower (Table 4).

## 4. Discussion

The number of ways that novel 3D-printable resins can be used in dentistry is growing quickly [12,13,14,19]. But to use it as a fixed dental prosthesis, you need a luting composite resin to make a bond that is stable and lasts a long time [18]. Printable ceramic–glass polymer materials are clinically promising because of the material savings and the ability to create more complex geometries compared to machinable ones. Different manufacturing methods may be effective in the bond strength of ceramic–glass polymers to the luting composite resin. The research showed that no significant difference was found between SBS of SLC to printable 3D-PR material, and to machinable ceramic–glass polymer materials (CS and VE). Therefore, the null hypothesis was not rejected.

There are many research studies to improve the bond quality of ceramic–glass polymer materials with different luting composite resins [3,4,5,8,9,30,35]. In this study, SLC was preferred because it is widespread and easy to use [9,22]. The present research focused on the effect of different manufacturing methods on the bond strength; improving the bond quality of ceramic–glass polymer materials was beyond the scope of this study, but future research should evaluate these.

Machinable ceramic–glass polymer materials are polymerized at higher rates (up to 96%) under high pressure and temperature, leaving few free monomers for copolymerization with luting composite monomers [36,37,38,39]. The lower degrees of conversion (76.11%) reported in the literature for 3D-PR might indicate better adhesion, but the results did not show this [40]. It should be taken into account that when a parameter is changed in the manufacturing or post-processing, the degree of conversion will be affected and also probably affect the bonding performance [41]. According to the literature [10,35,37,39], it has been suggested that micro-retentive surfaces be generated prior to luting by either airborne particle abrasion or hydrofluoric acid etching for VE and CS. According to the literature, 50 μm Al_2_O_3_ airborne particle abrasion resulted in better bond strength values [3,37,42]; 0.1–0.2 MPa is the recommended airborne particle abrasion pressure for machinable ceramic–glass polymers and is lower than the pressure prescribed for metal and ceramic restorations. Thus, 0.1 MPa was chosen because of concerns that airborne particle-abraded machinable ceramic–glass polymers at higher pressures would cause subsurface fractures [38]. Because previous researchers have shown that airborne particle abrasion for more than 30 s reduces bond strength for machinable ceramic–glass polymers, 15 s was used [24]. Therefore, the pretreatment with 0.4 MPa 50 μm Al_2_O_3_ airborne particle abrasion was reported to have the highest bond strengths for temporary printable resin [18]. It is generally believed that silane application increases bond strength following the formation of micro-retentive areas. However, there have been no consistent reports on the effectiveness of silane for bonding of ceramic–glass polymer materials, so silane application has not been used [36,43,44].

The shear test and micro-tensile test are the most common methods used to measure bond strength in the dental literature. Both advantages and disadvantages have been extensively discussed and explained in the past [27,32]. The SBS test has been reported to be the fastest and most convenient method of obtaining accurate results, and there is a good correlation between in vitro SBS data and clinical bond performance [27]. Although it is believed that the stress is more uniformly distributed during a micro-tensile test, specimen preparation is extremely challenging, and bond strength tends to increase with smaller bonding areas [45]. After a direct comparison of the various test results, the use of Weibull statistics has been proposed to provide more information because bond strength tests have low reliability [31,32].

According to Barutcugil et al. [9], the SBS of the SLC to VE specimens was reported to be 9.139 ± 2.428 MPa. In another study, the SBS of the SLC to the specimens was reported to be 12.69 ± 2.32 MPa for VE and 10.76 ± 2.23 MPa for CS [30]. The results of the current study are similar to other studies in the literature (the SBS of VE was 12.03 ± 2.11 MPa and CS was 11.02 ± 2.96 MPa). The SBS value of the newly introduced 3D-PR (9.74 ± 2.88 MPa) is slightly lower than the others. This may be because, unlike the pre-polymerized machinable blocks compared in the study, post-polymerization was required for printable 3D-PR. Mostafavi et al. [46] reported that post-processing procedures in AM affect the accuracy of the material being tested. Considering that the quality of post-processing depends on the operator, pre-polymerized machinable blocks are the more standard method. Although this study indicated that there was no significant difference in the ability to bond to SLC between machinable and printable ceramic–glass polymer materials, different post-processing procedures may alter the results. Future studies may be considered to compare the effect of different post-processing procedures on bond strength. However, SBSs of all tested materials in this study had enough to ensure good clinical performance, as a limit of 10–13 MPa is considered the minimum for acceptable clinical bond strength [47,48].

According to failure mode analysis, machinable and printable ceramic–glass polymers exhibited comparable failure modes in the present study. The predominant failure mode was a mixed failure, and there was no adhesive failure in all three groups. These findings were compatible with SBS values. Cohesive and mixed modes are preferable to adhesive failure mode because adhesive failure mode is typically related to low bond strength values [2,8,35]. In the study conducted by Sresthadatta et al. [30], adhesive failure was observed in all control specimens that had no surface treatment. Then, adhesive failure modes decreased and mixed-failure modes increased in surface treatment groups [30]. However, cohesive failure in the material is not a sign of a strong bond; rather, it may also be explained by the mechanics of the test and the brittleness of the components involved [45]. Barutcugil et al. [9] observed 50% adhesive, 40% mixed, and 10% cohesive failure in airborne particle-abraded VE specimens with macro-shear test (approximately 3 mm^2^). The increased mixed failure rates of the VE group (66.7%) may depend on an expanded bonded area (7.06 mm^2^) and the use of a notch-shaped rod in the present study. A notch-shaped rod was used instead of a knife-edge rod for testing in order to avoid inhomogeneous stress concentrations [45]. Nagasawa et al. [4] reported that the airborne particle-abraded CS group failed only cohesively within the ceramic using the macro-shear test (28.26 mm^2^). Concerns have been reported that the macro SBS test procedure results in cohesive failure of the substrate due to inhomogeneous stress distribution dependent on the expanded bonded area [9,27,31,32].

There is no single value to specify the strength of brittle materials such as resin composites, ceramics, and tooth structures due to the variability in the existence of strength-controlling defects in these [32]. Weibull statistics are suggested to both specify the strength of brittle materials and to improve the reliability and interpretation of bond strength tests [26,31,32,33]. According to Figure 3, both printable and machinable materials showed homogeneous distribution, and VE showed higher Weibull modulus compared to CS and 3D-PR, respectively. Unexpectedly, CS and 3D-PR showed similar slopes. This shows that the same types of defects are active in both sets of specimens. Unlike industrially polymerized CS and VE, 3D-PR resin has to undergo post-processing (such as cleaning with alcohol and post-curing) and appears to have been able to be achieved consistently, although complete polymerization is dependent on the operator. It is seen that the SBSs are parallel to the filler ratios of the substrates, respectively. 3D-PR was the less compliant material since it consists of 30–50 wt% of fillers, explaining its lower Weibull modulus with SLC (70 wt%). It appears that structural reliability increases when the luting agent and substrate are well-matched [31,49]. Nagasawa et al. [4] reported that the 70 μm Al_2_O_3_ airborne particle-abraded CS with another SLC (G-CEM one; GC) had a 4.8 ± 0.5 Weibull modulus. This Weibull modulus was similar with the present study’s result for CS (*m* = 4.22). Another study [26] examined the strength of the same SLC (RelyX U200; 3M) with dentin and enamel and found the Weibull modulus to be 5.2 and 6.7, respectively. With this information, it is possible to say that SLC has similar reliability on both sides of the adhesive sandwich containing the tooth and restoration. In addition, another issue to consider with SLCs is which light curing unit is preferred for polymerization, because the compatibility of the wavelength range of the light curing unit with photoinitiators is reported to affect bond strength [50].

The present study investigated the effect of different manufacturing methods on the SBS between ceramic–glass polymer materials and SLC using a simplified surface treatment with airborne particle abrasion. This study’s design has several limitations. The fact that the materials compared have different filler ratios makes it difficult to establish a direct relationship when comparing production methods, but the study is important in terms of comparing accessible production methods. As far as the authors are aware, no permanent 3D printing ceramic–glass polymer resin currently has a filler ratio above 50%. In the future, if 3D permanent resins with higher filler content are introduced to the market, the results must be updated. When the bond between the SLC and the restorative material was evaluated, no seating pressure was provided, and air bubbles as a function of thickness may have led to the failure (Figure 2b). In addition, the SBS of cohesive failure in luting composite was found to be significantly lower (Table 4). It is difficult to compare results to clinical situations because it does not replicate the oral environment. Nevertheless, in vitro studies still can serve for ranking the materials within the same conditions. Different test techniques can be used to determine the bond strength of materials. This study’s findings can be used for screening purposes, but they must be confirmed by additional research employing the micro-tensile and fracture toughness test. More studies are needed to evaluate the long-term durability of the new 3D-PR, including the aging process and thermal cycle, different adhesive systems, and different surface treatment strategies.

## 5. Conclusions

The following conclusions were reached within the limits of this study:

1. There is no difference in the SBS of the SLC to both printable 3D-PR and machinable ceramic–glass polymers, VE or CS. SBSs of all tested materials had enough to ensure good clinical performance.
2. The lowest Weibull modulus was seen for the printable 3D-PR, but both printable and machinable materials in the present study have shown a homogenous distribution.
3. In the failure modes of both printable and machinable materials in the present study, there is no difference, and the predominant failure mode was mixed mode for 3D-PR, VE, and CS.

## Figures and Tables

**Table 1:** Manufacturers and contents of the materials used in the study.

Material		Manufacturer	Composition	Lot Number
Machinable Blocks	(CS) Cerasmart 270	GC Dental Products, Leuven, Belgium	Organic part: Bis-MEPP, UDMA, DMA Inorganic part: 71 wt% silica (0.02 μm) and barium glass (0.3 μm) nanoparticles	2102176
	(VE) VITA Enamic	VITA Zahnfabrik, Bad Säckingen, Germany	Organic part: UDMA, TEGDMA Inorganic part: 86 wt% glass ceramic (SiO_2_, Al_2_O_3_, Na_2_O, K_2_O, and other oxides)	73340
Printable Resin	(3D-PR) VarseoSmile Crown Plus is distributed by Formlabs as Permanent Crown	Bego, Bremen, Germany	4′-isopropylidiphenol, ethoxylated and 2-methylprop-2enoic acid. Silanized dental glass, methyl benzoylformate, diphenyl (2,4,6-trimethylbenzoyl) phosphine oxide, 30–50 wt% inorganic fillers (particle size 0.7 μm)	600163
Self-adhesive luting composite	(SLC) RelyX™ U200	3M ESPE, St. Paul, MN, USA	Base paste: Silane-treated glass filler, 2-propenoic acid, 2-methyl 1,1′-[1-(hydroxymethyl)-1,2-ethanediyl] ester, triethylene dimethacrylate, sodium persulfate andper−3,5,5-trimethylhexanoate t-butyl. Catalyst paste: Silanated filler, dimethacrylate, silane-treated filler, sodium p-toluenesulfonate,1-benzyl-5-phenyl-baric acid, calcium salts, 1,12-dodecane dimethacrylate, calcium hydroxide and titanium dioxide (~70 wt% filler)	7784355

**Table 2:** Mean and standard deviation (SD) of the shear bond strength (MPa) with confidence intervals (95% CI) values of tested groups.

Material	N	Mean ± SD (MPa)	95% CI	*p*
3D-PR (Permanent Crown)	12	9.74 ± 2.88	[7.91; 11.58]	
VE (VITA Enamic)	12	12.03 ± 2.11	[10.68; 13.37]	0.129
CS (Cerasmart 270)	12	11.02 ± 2.96	[9.13; 12.90]	

ANOVA.

**Table 3:** Distribution of failure modes.

	Adhesive n (%)	Mixed n (%)	Cohesive-Material n (%)	Cohesive-Luting Composite n (%)	*p*
3D-PR (Permanent Crown)	0 (0)	7 (58.3)	2 (16.7)	3 (25)	0.986
VE (VITA Enamic)	0 (0)	8 (66.7)	2 (16.7)	2 (16.7)	
CS (Cerasmart 270)	0 (0)	7 (58.3)	2 (16.7)	3 (25)	

Chi-Square test; *p* < 0.05.

**Table 4:** Comparing the mean shear bond strength (MPa) of failure modes.

Failure Mode	Mean ± SD (n)	*p*
Adhesive	- (0)	
Cohesive-Material	11.1 ± 3 (6)	0.033
Cohesive-luting composite	8.9 ± 1.6 (8)	
Mixed	11.6 ± 2.7 (22)	

Kruskal–Wallis; *p* < 0.05.

## Data Availability

The raw data supporting the conclusions of this article will be made available by the authors on request.
